# Complete Blood Count and Retinal Vessel Calibers

**DOI:** 10.1371/journal.pone.0102230

**Published:** 2014-07-18

**Authors:** Gerald Liew, Jie Jin Wang, Elena Rochtchina, Tien Yin Wong, Paul Mitchell

**Affiliations:** 1 Centre for Vision Research, University of Sydney, Sydney, New South Wales, Australia; 2 Centre for Eye Research Australia, University of Melbourne, Melbourne, Victoria, Australia; 3 Singapore Eye Research Institute, Yong Loo Lin School of Medicine, National University of Singapore, Singapore, Singapore; Queen's University Belfast, United Kingdom

## Abstract

**Objective:**

The influence of hematological indices such as complete blood count on microcirculation is poorly understood. Retinal microvasculature can be directly visualized and vessel calibers are associated with a range of ocular and systemic diseases. We examined the association of complete blood count with retinal vessel calibers.

**Methods:**

Cross-sectional population-based Blue Mountains Eye Study, n = 3009, aged 49+ years. Complete blood count was measured from fasting blood samples taken at baseline examination, 1992–4. Retinal arteriolar and venular calibers were measured from digitized retinal photographs using a validated semi-automated computer program.

**Results:**

All analyses adjusted for age, sex, systolic blood pressure, diabetes, smoking and fellow vessel caliber. Higher hematocrit, white cell count and platelet count were associated with narrower arteriolar caliber (p = 0.02, 0.03 and 0.001 respectively), while higher hemoglobin, hematocrit, red cell count, white cell count and platelet count were associated with wider venular caliber (p<0.0001 for all). Each quintile increase in hematocrit, white cell count and platelet count was associated with approximately 0.5 µm narrower arteriolar caliber; whereas each quintile increase in all of the complete blood count components was associated with approximately 1–2 µm wider venular caliber.

**Conclusions:**

These associations show that elevated levels of hematological indices can have adverse effects on the microcirculation.

## Introduction

The influence of hematological indices such as the complete blood count on microvasculature is poorly understood. Given the close physical proximity of red cells, leucocytes and platelets to the vessel endothelium, a relationship between these indices and microvascular changes would be expected. For example, an increase in blood components such as hematocrit or red cell count may be accompanied by an increase in circulating blood volume,[1] which would be expected to be associated with wider venular calibers.

The retinal vasculature is one of the few microcirculatory systems that can be directly visualized. Methods have been developed that allow accurate measurement of retinal vessel calibers from digitized retinal photographs.[2] Retinal vessel calibers are associated with a range of systemic cardiovascular risk factors and outcomes, with different associations found for arteriolar and venular calibers. For example, narrower arteriolar caliber is an adverse marker of microcirculatory health, and is associated with elevated blood pressure [3], [4], greater body mass index [5], and endothelial dysfunction.[6], [7] In contrast, wider retinal venular caliber is a marker of hyperglycemia,[8], [9] cerebral hypoxia,[10] endothelial dysfunction, obesity[11] and systemic inflammation.[5], [7] Studying these retinal vascular caliber changes provides insight into parallel pathology that may occur in the systemic micro- and macro-circulations, with retinal arteriolar narrowing strongly associated with angiographically defined coronary artery occlusion,[12] reduced myocardial perfusion,[13] and greater aortic stiffness. [14] Wider venules are associated with chronic kidney disease,[15], [16] aortic calcification[17] and progression of cerebral small vessel disease.[18] Among people with diabetes, wider venular caliber is directly related to the presence and severity of diabetic retinopathy.[19], [20]

Klein et.al recently reported from the Beaver Dam Eye Study (BDES)[21] that the components of the complete blood count (red cell count, hemoglobin, hematocrit and white cell count, and to a lesser extent platelet count) are directly associated with wider retinal arteriolar and venular calibers. These associations persisted after adjustment for age, blood pressure and other confounders. However, when the effect of fellow vessel caliber was taken into account, the association of hematocrit, haemoglobin and red blood cell count with arteriolar caliber (but not venular caliber) reversed, while that of platelet count became nonsignificant. [22] This may suggest a degree of confounding. Few other studies have addressed this issue. We therefore aimed to examine these associations of complete blood count components with retinal arteriolar and venular calibers in another independent sample, a population-based study of older individuals.

## Materials and Methods

The Blue Mountains Eye Study (BMES) is a population-based study of vision and eye disease in an urban Australian Caucasian population aged 49 years or older, full details of which are reported elsewhere [23]. Briefly, at baseline 3654 (82.4%) of 4433 eligible residents living in two post-code areas of the Blue Mountains, west of Sydney, were examined during 1992–4. Of these, 299 were excluded due to ungradable photographs for retinal vessel calibers, and a further 346 without data on complete blood count, leaving 3009 participants who contributed data to this analysis. The Human Ethics Committees of the Western Sydney Area Health Service and University of Sydney approved all three examinations. Signed informed consent was obtained from participants at each examination.

**Figure 1:**
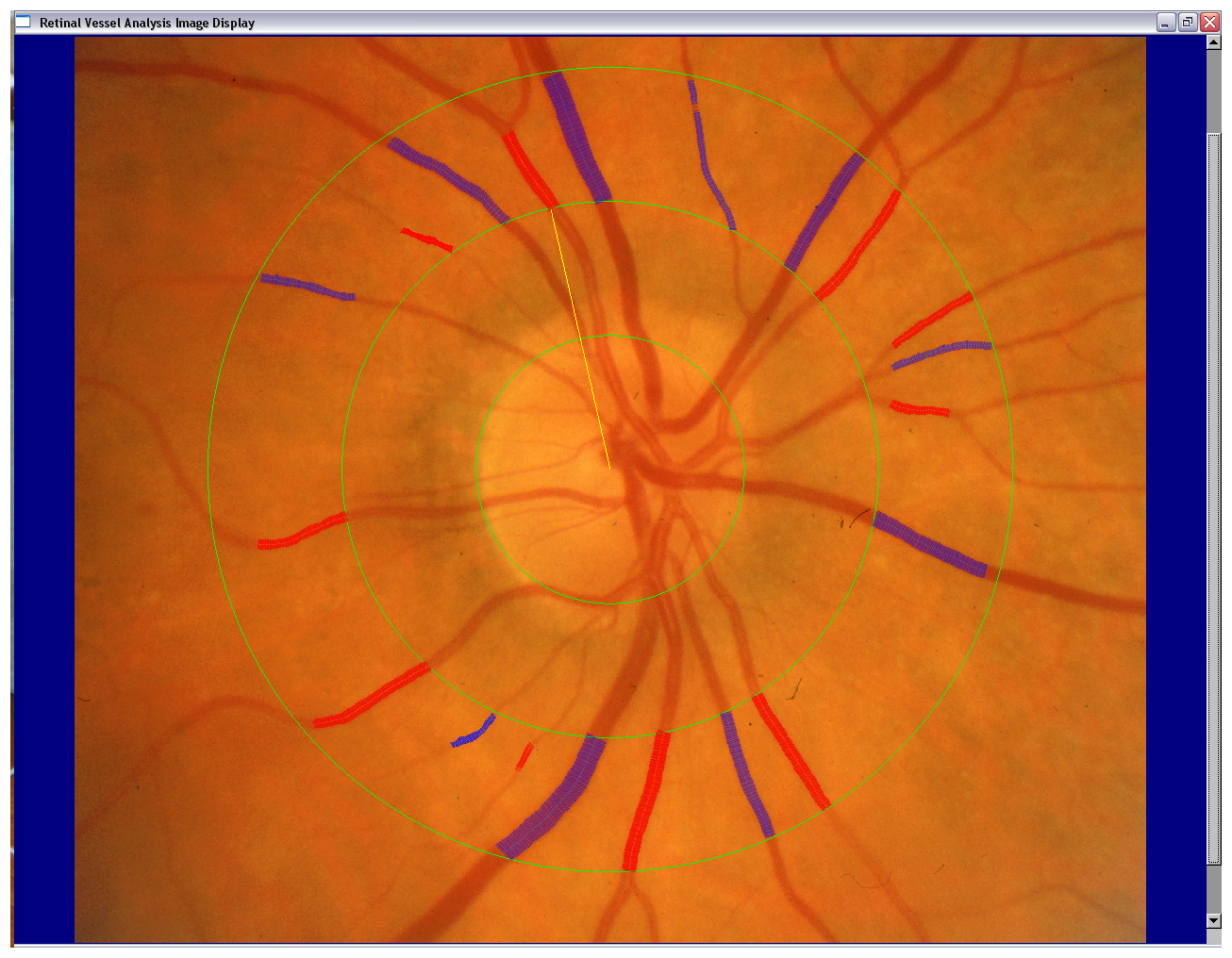
Measurement of retinal arterioles (red lines) and venules (blue lines) passing through a region between ½ to 1 disc diameters from the optic disc margin.

At the baseline examination, 30 degree stereoscopic retinal photographs of the macula and 5 other retinal fields of both eyes were taken using a Zeiss FF3 fundus camera (Carl Zeiss, Oberkochen, Germany). [24] A computer-assisted grading method with high reproducibility was used to measure retinal vessel caliber[25]. In brief, digitised retinal images of one eye (mainly right eyes) of each participant were displayed and all vessels greater than 25 µm in diameter and completely passing through the region between ½ to 1 disc diameter from the optic disc margin were measured.(Figure 1) Central retinal arteriolar and venular equivalents (representing average arteriolar and venular calibers) were calculated using the Parr-Hubbard formula[2]. Intra- and inter-grader grading reliability of this method is good [25], with quadratic weighted kappa 0.85 for arteriolar measurements and 0.90 for venular measurements for inter-grader reliability and between 0.80–0.93 for intra-grader reliability.

Fasting blood samples were collected from participants and the Institute of Clinical Pathology and Medical Research at Westmead Hospital performed laboratory tests within 4 hours of blood collection. Hemoglobin was measured on a Technician H2 haematology analyser, hematocrit by spun microhematocrit, red and white blood cell count using Coulter Counter methods (Beckman Coulter, Inc., Fullerton, California).

At baseline, blood pressure was measured after participants had been comfortably seated using the same mercury sphygmomanometer with appropriate cuff size. Smoking status was defined from history as never smoked, ex-smoker and current smoker (which included those who had ceased smoking within the last 12 months). We measured fasting blood sugar and fasting total cholesterol and defined diabetes as a physician diagnosis of diabetes, or a fasting blood sugar ≥7 mmol/L.

Statistical analyses were performed using SAS version 9 (SAS Institute Inc., Cary, NC). Due to the influence of gender on the distribution of components of the complete blood count, we analyzed each blood component based on sex-specific quintiles i.e. the quintiles for each blood component were computed for each sex initially, then combined to obtain the combined quintile for the entire group. Mean arteriolar and venular caliber was compared in each quintile of complete blood count component using analysis of variance. Linear regression models were constructed adjusting for age and gender (model 1), model 1 variables and systolic blood pressure, diabetes, smoking (model 2), and model 2 parameters and either arteriolar or venular caliber of the same eye. Partial R^2^ were used to estimate the amount of variance explained by the blood component being analyzed.

## Results

The mean age of the 3009 included participants was 65.5 years, compared to 69.5 years for the 645 excluded participants. Other than being older, and more likely to be current or never smokers, excluded participants were similar to included participants in terms of gender, blood pressure, diabetes and complete blood count parameters. (Table 1).

**Table 1:** Included and excluded participants.

Risk Factor	Included N = 3009	Excluded N = 645	p-value
Age (years)	65.5 (9.2)	69.5 (11.5)	<0.0001
Systolic blood pressure (mmHg)	146.1 (21.3)	146.6 (22.9)	0.59
Hematocrit (%)	14.9 (1.3)	14.7 (1.5)	0.10
Hemoglobin (g/dL)	0.44 (0.04)	0.43 (0.05)	0.15
Red cell count (10^6^/µL)	4.8 (0.4)	4.8 (0.5)	0.09
White cell count (10^3^/µL)	6.5 (1.8)	6.7 (1.9)	0.11
Platelet count (10^3^/µL)	262.7 (63.4)	259.8 (68.1)	0.52
Female	57.0	55.2	0.39
Diabetes	7.9	7.1	0.45
Current smoker	14.6	16.1	
Ex-smoker	35.6	29.3	
Never-smoker	49.8	54.6	0.01

Numbers are means (standard deviations) or %.

Each increasing quintile of hemaglobin, hematocrit, red cell count and white cell count, but not platelet count, was associated with wider arteriolar caliber. (p for trend 0.001, 0.003, 0.0006 and 0.03 respectively (Table 2). Higher values of all these blood components were associated with wider venular caliber (p for trend <0.0001 for all components, Table 2).

**Table 2:** Blood components and unadjusted retinal arteriolar and venular calibers.

				Arteriolar caliber (µm)				Venular caliber (µm)			
**Blood Element**	Gender		Range	N	Mean	SD	p-value for trend	N	Mean	SD	p-value for trend
**Hemoglobin**	Q1	W	8.4–13.5	631	188.6	21.1	0.001	631	217.1	19.9	<0.0001
**g/dL**		M	7.6–14.7								
	Q2	W	13.6–14.0	531	189.7	20.7		531	220.6	19.4	
		M	14.8–15.3								
	Q3	W	14.1–14.5	644	190.0	19.0		644	222.2	19.1	
		M	15.4–15.9								
	Q4	W	14.6–15.1	604	191.0	20.1		604	221.5	19.8	
		M	16.0–16.4								
	Q5	W	15.2–22.4	599	193.2	20.8		599	228.6	21.1	
		M	16.5–19.3								
**Hematocrit**	Q1	W	0.27–0.39	521	189.5	21.0	0.003	521	217.6	19.7	<0.0001
**%**		M	0.22–0.42								
	Q2	W	0.40–0.41	686	189.6	20.3		686	219.4	19.1	
		M	0.43–0.44								
	Q3	W	0.42–0.42	545	189.2	19.4		545	222.2	19.8	
		M	0.45–0.46								
	Q4	W	0.43–0.44	735	190.9	20.1		735	222.2	20.1	
		M	0.47–0.48								
	Q5	W	0.45–0.66	522	193.5	21.1		522	229.2	21.0	
		M	0.49–0.56								
**Red cell count**	Q1	W	3.25–4.39	591	188.2	20.9	0.0006	591	217.4	19.9	<0.0001
**10^6^/µL**		M	2.19–4.70								
	Q2	W	4.40–4.59	600	189.3	20.4		600	219.3	19.3	
		M	4.71–4.95								
	Q3	W	4.66–4.77	613	190.3	19.1		613	221.9	19.5	
		M	4.96–5.17								
	Q4	W	4.78–4.98	602	191.8	20.0		602	223.6	19.9	
		M	5.18–5.37								
	Q5	W	4.99–6.35	603	192.8	21.2		603	227.6	21.0	
		M	5.38–6.85								
**White cell count**	Q1	W	2.3–5.0	618	191.1	19.4	0.03	618	219.9	18.9	<0.0001
**10^3^/µL**		M	2.7–5.2								
	Q2	W	5.1–5.7	586	188.7	20.0		586	219.7	18.9	
		M	5.3–6.0								
	Q3	W	5.8–6.5	625	190.4	20.6		625	221.0	19.6	
		M	6.1–6.8								
	Q4	W	6.6–7.5	567	189.8	20.4		567	222.6	20.5	
		M	6.9–7.8								
	Q5	W	7.6–18.7	610	192.4	21.4		610	226.6	22.3	
		M	7.9–17.6								
**Platelet count**	Q1	W	108–223	597	191.1	19.8	0.30	597	219.8	19.7	<0.0001
**10^3^/µL**		M	106–198								
	Q2	W	224–253	609	189.1	21.9		609	220.3	20.1	
		M	199–227								
	Q3	W	254–284	603	190.2	20.8		603	221.9	20.3	
		M	228–255								
	Q4	W	285–321	596	190.8	19.7		596	223.8	20.1	
		M	256–288								
	Q5	W	322–636	596	191.5	19.7		596	224.3	20.4	
		M	289–588								

M refers to Men, W refers to Women. Q1–5 refers to quintiles. SD to standard deviation.

In analyses adjusted for age, sex and cardiovascular risk factors, higher hemoglobin, hematocrit and red cell count remained associated with wider arteriolar caliber. (Table 3) White cell count and platelet count levels were not associated with arteriolar caliber. After additional adjustment for venular caliber, however, higher levels of haemoglobin, hematocrit, white cell count and platelet count became associated with narrower arteriolar caliber, suggesting a confounding effect of venular caliber. Red cell count was no longer associated with arteriolar caliber after adjusting for venular caliber. The contribution to the variation of arteriolar caliber (reductions in partial R^2^) by each of these blood components in all models remained small, at 0.001–0.004.

**Table 3:** Multivariable adjusted arteriolar and venular calibers.

	Arteriolar caliber (µm)								
	Model 1			Model 2			Model 3		
**Blood element**	Beta Estimate*	P-value	Partial R^2^ **	Beta Estimate*	P-value	Partial R^2^ **	Beta Estimate*	P-value	Partial R^2^ **
**Hemoglobin**	0.64	0.01	0.002	0.54	0.03	0.001	−0.39	0.06	0.001
**Hematocrit**	0.65	0.01	0.002	0.52	0.04	0.001	−0.51	0.02	0.001
**Red cell count**	0.74	0.003	0.003	0.91	<0.001	0.004	−0.20	0.36	0.002
**White cell count**	0.51	0.04	0.001	0.14	0.56	<0.001	−0.47	0.03	0.001
**Platelet count**	−0.20	0.43	<0.001	−0.27	0.28	<0.001	−0.68	0.001	0.002
	Venular caliber (µm)								
**Hemoglobin**	2.1	<0.0001	0.10	1.8	<0.0001	0.02	1.5	<0.0001	0.01
**Hematocrit**	2.3	<0.0001	0.02	2.0	<0.0001	0.02	1.7	<0.0001	0.01
**Red cell count**	2.1	<0.0001	0.02	2.2	<0.0001	0.02	1.7	<0.0001	0.02
**White cell count**	1.7	<0.0001	0.01	1.2	<0.0001	0.01	1.1	<0.0001	0.01
**Platelet count**	0.9	0.0004	0.004	0.8	0.001	0.003	0.9	<0.0001	0.004

Model 1 is adjusted for age and sex; Model 2 adjusted for variables in Model 1 and systolic blood pressure, diabetes, smoking; Model 3 adjusted for variables in model 2 and either venular or arteriolar caliber.

P values are for trend.

* per quintile increase in blood count component.

** for blood count component in the given model.

Higher levels of hemoglobin, hematocrit, red cell count, white cell count and platelet count remained associated with wider retinal venular caliber, after adjustment for age, gender, cardiovascular risk factors and arteriolar caliber (Table 3). Each increased quintile of blood component was associated with 1–2 µm wider venular caliber, with relatively greater contribution to the variation of venular caliber than to arteriolar caliber: partial R^2^ reduction by 0.01–0.02 in the final model 3 for hemoglobin, hematocrit red cell count and white cell count, and by 0.004 for platelet count.

We performed additional analyses modelling blood components as continuous variables in model 3. These analyses showed the same results as those with quintiles of blood components.

## Discussion

Retinal arteriolar and venular calibers are associated with systemic cardiovascular parameters such as blood pressure and inflammatory markers.[17], [26], [27] We found that in a population of older individuals, higher values of most components of the complete blood count (hemoglobin, hematocrit, white cell count and platelet count) were associated with narrower retinal arteriolar caliber after adjustment for factors such as age, blood pressure, smoking, diabetes and venular caliber. All of these haematological measures, as well as red cell count, were associated with wider retinal venules.

Our results are similar to those of the BDES for venular caliber, and only slightly different for arteriolar caliber.[21] The BDES reported each quintile increase in hemoglobin, hematocrit and red cell count was associated with approximately 3 µm wider venular caliber, while our study found the increase to be approximately 2 µm. For white cell count and platelet count, the corresponding widening in venular caliber was 1.0 µm and 0.9 µm, respectively, in the BDES, and 1.2 µm and 0.8 µm, respectively, in our study. These results were similar for analyses excluding and including the effect of fellow vessel caliber. With regard to arteriolar caliber, both the BDES and our study found higher levels of hemoglobin, hematocrit, and red blood cell count were directly associated with wider arteriolar caliber before adjusting for the effects of fellow vessel caliber. However, after additional adjustment for fellow vessel caliber, which we believe provides a more fully adjusted estimate, the relationship of these parameters with arteriolar caliber reversed, and all except red blood cell count were now associated with narrower arteriolar caliber (approximately 0.5 µm narrower, after adjustment for venular caliber). After fellow vessel adjustment higher red blood cell count was associated with narrower arteriolar caliber in the BDES, but not in our study, while higher platelet count was associated with narrower arteriolar caliber in our study but not in the BDES [21]. Both studies showed an association of white cell count with narrower arteriolar caliber after fellow vessel adjustment. In all cases, the contribution of the blood parameter to explaining variation in retinal vessel caliber was lower for arterioles (0.1–0.2%) than for venules (1–2%). Our results are therefore mostly concordant with those of the BDES except for platelet and red cell count, where we found platelet count but not red cell count was associated with arteriolar caliber, whereas the BDES found the converse. Possible reasons for the different findings are random chance, and the fact that our population was somewhat older than the BDES (mean age 65.5 years versus 61.7 years) and had a higher mean systolic blood pressure (146.1 mmHg versus 132.0 mmHg) which may have reduced the ability of arterioles to respond to changes in red cell count. The BDES report suggested that future analyses of vessel caliber may need to take into account complete blood count markers; [21] our results suggest that this may be useful if such data are available, but is not essential given the small contribution of blood parameters to variation in vessel caliber.

There are a number of other studies with which to compare our results. In patients with AIDS, lower hematocrit is associated with wider retinal arteriolar caliber,[28] while patients with chronic anemia from sickle cell disease have coronary artery dilation.[29] Experimentally induced anemia in lambs likewise causes cerebral artery dilation.[30] These findings showing an inverse association between hematocrit and arteriolar and arterial caliber are consistent with our results. Venular changes with hematocrit are poorly studied, but a report that sickle cell anemia is associated with reduction in conjunctival vessel caliber[31] supports our findings. Persons living at higher altitudes tend to have higher hematocrit and a study which experimentally lowered hematocrit by transferring volunteers from Bolivian highlands to a lower altitude found transiently dilated retinal venules in the volunteers, though these returned to normal caliber after 2.5 months. [32] Early reports of fundal changes of arterial narrowing and venular dilation in polycythemia rubra vera corroborate our findings.[33] The dilation of retinal arterioles with decreasing hematocrit and hemoglobin may be a compensatory mechanism to lower vascular resistance and increase blood flow, in response to reduced oxygen carrying capacity.[34]

Elevated white cell count is consistently associated with wider retinal venular caliber in several population studies,[17], [26], [27] and clinically, chronic leukemia may present with dilated retinal venules.[35] Leucocytosis is associated with increased aortic arch atheroma,[36] which is also associated with wider retinal venules,[17] further highlighting the influence of this blood parameter on both maco- and microcirculation. This association has been attributed to systemic inflammation, but a direct leucocytic influence on endothelium may also play a role.[27], [37] Such leukocyte-endothelium interactions may also underlie the narrower arterioles we observed with leukocytosis.

The influence of platelet count on microvascular caliber is largely unknown. We observed differential effects of increased platelet count in arterioles and venules. Platelets affect arteriolar endothelial function through pathways such as P-selectin,[38] which may account for the association with arteriolar narrowing. The distribution of platelet flow,[38] and possibly the expression of Von Willebrand factor under certain conditions,[39] differs in venules from that in arterioles, which may explain the differential associations with raised platelet count in these two small vessel types.

The biochemical mechanisms underlying the associations we observed are unclear and the literature on the topic is sparse. We can speculate that the potent endothelium derived vasodilator nitric oxide (NO) is involved, as oxyhemoglobin is a strong and irreversible NO scavenger,[40] and increased levels of haemoglobin, hematocrit and red cell count may increase the amount of NO scavenged and reduce endothelial mediated vasodilation, leading to narrower arteriolar caliber. White cells and platelets also release cytokines that modulate the balance between pro and antioxidant molecules, which has a major effect on vascular remodelling[41], [42] and vessel calibers. This is an area that warrants further research, particularly as the changes we have described are known to be associated with increased risk of cardiovascular events.[43]

Strengths of this study include its large sample size and ability to adjust for important confounders. Our study is limited by its cross-sectional nature, which prevents inferences about the temporal sequence of associations and may not apply to prospective data. We adjusted for the fellow vessel caliber to control for the correlation or shared variances of the two vessel types [26] as other researchers have done.[21] This method may over adjust if shared genetic factors are to be investigated. As this study did not examine such shared factors, this limitation is of less concern. Finally there may be measurement errors in vessel caliber which may have influenced our results, although these would be non-differential and expected to reduce any associations we observed.

In summary, we found that higher hemoglobin, hematocrit, white cell count and platelet count are associated with narrower retinal arterioles, while higher levels of all complete blood components (the above as well as red cell count) are associated with wider retinal venules. These associations show that elevated levels of readily measured hematological indices can have adverse effects on the microcirculation and may need to be considered in studies of retinal vessel blood flow.

## Conclusions

The influence of hematological indices such as complete blood count on microcirculation is poorly understood. We report that higher hematocrit, white cell count and platelet count were associated with narrower retinal arteriolar caliber (p = 0.02, 0.03 and 0.001 respectively), while higher hemoglobin, hematocrit, red cell count, white cell count and platelet count were associated with wider retinal venular caliber (p<0.0001 for all). These associations show that elevated levels of readily measured hematological parameters can have adverse effects on the microcirculation and may need to be considered in studies of microvascular blood flow.
